# Hepato-pancreato-biliary emergencies for the acute care surgeon: etiology, diagnosis and treatment

**DOI:** 10.1186/s13017-015-0004-y

**Published:** 2015-03-08

**Authors:** Jean M Butte, Morad Hameed, Chad G Ball

**Affiliations:** Department of Surgery, Foothills Medical Center, University of Calgary, Calgary, AB Canada; University of British Columbia, Vancouver, BC Canada

**Keywords:** Emergency surgery, Hepatopancreatobiliary, Acute care surgeon, Pancreatitis, Acute cholecystitis, Acute cholangitis, Surgery, Initial treatment

## Abstract

Hepatopancreatobiliary (HPB) emergencies include an ample range of conditions with overlapping clinical presentations and diverse therapeutic options. The most common etiologies are related to cholelithiasis (acute cholecystitis, pancreatitis, and cholangitis) and non-traumatic injuries (common bile duct or duodenal). Although the true incidence of HPB emergencies is difficult to determine due to selection and reporting biases, a population-based report showed a decline in the global incidence of all severe complications of cholelithiasis, primarily based on a reduction in acute cholecystitis. Even though patients may present with overlapping symptoms, treatment options can be varied. The treatment of these conditions continues to evolve and patients may require endoscopic, surgical, and/or percutaneous techniques. Thus, it is essential that a multidisciplinary team of HPB surgeons, interventional gastroenterologists and radiologists are available on an as needed basis to the Acute Care Surgeon. This focused manuscript is a contemporary review of the literature surrounding HPB emergencies in the context of the acute care surgeon. The main aim of this review is to offer an update of the diagnosis and management of HPB issues in the acute care setting to improve the care of patients with potential HPB emergencies.

## Introduction

Hepatopancreatobiliary (HPB) emergencies for the Acute Care Surgeon (ACS) include a wide range of diseases with variable clinical presentations and diverse therapeutic options. Fortunately most acute presentations can be classified as consequences of either inflammatory (gallstones, pancreatitis, cholangitis) or injury (common bile duct, duodenum) etiologies [1-3].

It is estimated that 1% to 4% of asymptomatic or mildly symptomatic patients will develop an acute complication of cholelithiasis [4]. These include cholecystitis, cholangitis and pancreatitis. Acute cholecystitis is the most frequent of these diseases [1,5]. Unfortunately determining the true incidence of HPB emergencies remains difficult due to selection and reporting biases. A recent population-based study showed a decline in the global incidence of all severe complications of cholelithiasis [6]. This was primarily based on a reduction in acute cholecystitis due to the wide spread adoption of laparoscopic cholecystectomy. Conversely, the incidence of acute biliary pancreatitis and cholangitis has increased during the same interval [6,7].

Although many of these HPB emergencies present with overlapping symptoms, treatment options can be diverse [8]. They may require endoscopic, surgical, and/or percutaneous techniques. As a result, it is essential that a multidisciplinary team of HPB surgeons, interventional gastroenterologists and radiologists are available on an as needed basis to the Acute Care Surgeon. Similarly, those patients who are not able to receive complete or gold standard therapy should be referred elsewhere without delay.

The aim of this review is to update the management of patients diagnosed with non-trauma HPB emergencies in the context of the ACS service.

### General assessment

Similar to trauma, the initial evaluation of patients presenting with a HPB emergency should include simultaneous diagnosis and therapy. This concurrent rapid assessment and treatment is particularly important for patients who present with sepsis. A detailed clinical history of the acute event, including a focused past medical history (i.e. history of gallstones, pancreatitis, duodenal ulcer/NSAID use, and/or cancer) and complete physical examination are crucial. These details may suggest the likely diagnosis, determine the severity of the acute event, and guide both immediate and subsequent treatments. It is important to note that most patients present with an inflammatory and/or septic complication of a previously known disease, as opposed to a completely de novo etiology. Thus, patients presenting with acute cholecystitis or another complication of gallstones typically have a known history of symptomatic cholelithiasis. By contrast, patients suffering from pancreatic diseases generally develop symptoms after an acute new event.

The first step in caring for these patients requires a direct assessment of the severity of the acute event itself. Septic shock and acute bleeding represent the most common causes of hemodynamic compromise and must be addressed immediately. These methodologies include intravenous fluid resuscitation, early initiation of antimicrobial therapy, and blood product transfusion as needed. It is important to highlight that these patients often present with nausea and vomiting, dehydration, acute kidney injury, electrolyte imbalances, anemia, and/or coagulation abnormalities. A recent systematic review of 78 trials showed that resuscitation with colloids does not reduce the risk of death, nor improve survival [9]. It is also more expensive than resuscitation with crystalloids [9]. Another prospective randomized trial showed that patients with severe sepsis undergoing resuscitation with Ringer’s solution had a decreased 90-day mortality and renal-replacement therapy rate when they were compared with patients who received Hydroxyethyl starch 130/0.42 [10]. Thus, it is recommended that patients with HPB emergencies undergo active and early resuscitation with crystalloids [9].

Numerous scoring models have been created to determine the severity of the acute HPB event [11]. These include, but are not limited to, the acute and physiology and chronic health evaluation (APACHE) III and IV, the simplified acute physiology score (SAPS) 3, and the mortality probability model (MPM) III. These scores were recently compared in 2596 patients with the aim of determining their value in predicting mortality [12]. The discriminatory performances of APACHE III and IV were similar, but also superior to both SAPS 3 and MPM III scores. Despite superior calibration and discrimination amongst surgical patients in particular, the APACHE III or IV assessments are not overly simple to determine in the initial resuscitation phase. It is also clearly important to obtain focused liver and pancreatic function, as well as liver enzyme tests during the evaluation to guide resuscitation and narrow the differential diagnosis.

Once effectively resuscitated, patients should undergo diagnostic imaging tests to rapidly determine the etiology and guide further treatment. The type of study required will depend on patient status and clinical suspicion. These may include bedside and/or formal abdominal ultrasound (US), hepatobiliary scintigraphy (HIDA), computed tomography (CT), endoscopic retrograde cholangiography (ERCP), magnetic resonance (MR) cholangiography and/or endoscopic ultrasound (EUS).

### Biliary inflammatory diseases

#### Acute calculous cholecystitis (AC)

Acute inflammation of the gallbladder is a frequent complication of cholelithiasis, and affects up to 20% of patients with recurrent symptomatic gallstones [4]. Despite the history that most patients provide (previous episodes of transient colic pain in their right upper quadrant), acute presentations to a health care facility are typically longer and associated with additional symptoms (nausea or vomiting after ingesting high fat foods). Obstruction of the cystic duct by a gallstone or sludge produces dilation of the gallbladder and increases its internal pressure [8]. Subsequent biliary stasis and the proliferation of microorganisms is typical. If the obstruction persists, venous outflow decreases, with dilatation of capillaries and lymphatics resulting in gallbladder wall edema and thickening [4]. Eventually the gallbladder develops areas of hemorrhage and necrosis due to vascular occlusion. Imaging and exploration may reveal both fluid and air within the gallbladder wall. If the ischemia and necrosis is located within the posterior wall (i.e. apposed to the liver), a pericholecystic abscess eroding into the liver commonly occurs. It is also important to note that specific complications such as perforation, biliary peritonitis, pericholecystic abscess and biliary fistula (between the gallbladder and duodenum, colon, or stomach) may alter the clinical presentation and increase morbidity and mortality of the disease. Bouveret’s syndrome, gastric outlet obstruction, biliary ileus and gallstone-related small bowel obstruction are uncommon complications that should be identified.

The initial assessment of these patients includes 2 dominant objectives: [1] confirming the diagnosis and [2] establishing its severity. Despite a wide array of options (US, HIDA, CT, MR), the revised Tokyo consensus guidelines represent the best parameters for directing diagnosis and treatment [5,13]. Based on diagnostic sensitivities of 90% to 95%, abdominal US remains the initial modality of choice [4]. Because it can be performed by the ACS within the emergency department itself, it is also cost saving and rapid. Identification of gallbladder wall thickening (>5 mm), an obstructing gallstone in the gallbladder neck, pericholecystic fluid, US Murphy’s sign and/or dilation and thickening of the common bile duct (CBD) are important signs that contribute to defining the diagnosis. Unfortunately CT imaging is far less specific and frequent in confirming this diagnosis. After confirmation however, the disease should be classified according its severity (grade I = mild, II = moderate, III = severe). While grade II refers to the presence of systemic signs of inflammation, grade III cholecystitis includes dysfunction of at least one organ/system [13] (Table 1).

**Table 1:** **“Severity assessment criteria for acute cholecystitis”**

**Grade**	**Definition**
I (mild)	Acute cholecystitis does not meet the criteria of “Grade III” or “Grade II”
	It can also be defined as acute cholecystitis in a healthy patient with no organ dysfunction and mild inflammatory changes in the gallbladder, making cholecystectomy a safe and low-risk operative procedure.
II (moderate)	Acute cholecystitis is associated with any one of the following conditions:
	1. Elevated white blood cell count (>18,000/mm3)
	2. Palpable tender mass in the right upper abdominal quadrant
	3. Duration of complaints > 72 hours
	4. Marked local inflammation (gangrenous cholecystitis, pericholecystic abscess, hepatic abscess, biliary peritonitis, emphysematous cholecystitis).
III (severe)	“Grade III” (severe) acute cholecystitis is associated with dysfunction of any one of the following organs/systems
	1. Cardiovascular dysfunction defined as hypotension requiring treatment with dopamine ≥ 5 μg/kg per min, or any dose of norepinephrine
	2. Neurological dysfunction defined as decreased level of consciousness
	3. Respiratory dysfunction defined as a PaO2/FiO2 ratio < 300
	4. Renal dysfunction defined as oliguria, creatinine > 2.0 mg/dl
	5. Hepatic dysfunction defined as PT-INR > 1.5
	6. Hematological dysfunction defined as platelet count < 100,000/mm3

From “Yokoe M, et al. [1] (with permission).

The treatment of patients with AC should include general medical therapy (nil per mouth (NPO), intravenous fluids, antibiotics and analgesia) followed by urgent cholecystectomy. The 2 dominant surgical issues include the type (open vs. laparoscopic) and timing (early vs. delayed) of the procedure. Two small prospective randomized trials compared open with laparoscopic surgery for acute cholecystitis. The first study showed that open cholecystectomy had a significantly higher number of postoperative complications, as well as a longer postoperative hospital stay (6 vs. 4 days). No mortality or bile duct injuries were observed in this study [14]. A more recent trial included 70 patients and did not show any significant difference in the rate of postoperative complications [15]. The laparoscopic group had a significantly longer median operating time (90 vs. 80 minutes) and shorter median postoperative stay. The timing of cholecystectomy has also been evaluated in prospective randomized trials. Numerous small studies have observed that patients undergoing early cholecystectomy have a shorter hospital stay, without any other significant differences [16-22]. A recent meta-analysis that included 451 patients from 5 trials comparing early (less than 7 days from the onset of symptoms) with delayed (more than six weeks after the index admission) cholecystectomy revealed no statistically significant difference between the groups with regard to bile duct injuries (BDI) or conversion to open surgery [23]. The hospital stay was 3 days shorter in the early group however. Importantly, 40 (17.5%) patients in the delayed group required an emergency cholecystectomy during their waiting period for non-resolving or recurrent AC. It is also evident from large population based studies that the rate of BDI increases with higher grades of cholecystitis [4]. The most recent prospective multicenter trial comparing the optimal timing for cholecystectomy (early, during the first 24 hours vs. delayed) in patients with acute cholecystitis confirmed that early cholecystectomy was associated with significantly lower morbidity (11.8 vs. 34.4%). Although conversion to open surgery, nor mortality differed between groups, the mean length of hospital stay (5.4 vs. 10 days) and hospital costs were significantly lower in the group treated with early cholecystectomy [24]. Taken together, these results suggest that early laparoscopic surgery should be considered the treatment of choice.

In summary, patients with grade I or II acute cholecystitis should undergo early laparoscopic cholecystectomy, with awareness of the extent of the gallbladder’s inflammation (i.e. defining biliary anatomy to prevent BDI). More specifically, the ACS surgeon must be particularly wary of the inflamed gallbladder that is contracted into the liver bed because the anatomy in this scenario represents the most common etiology for BDI. Patients with grade III acute cholecystitis should undergo cholecystectomy once organ dysfunction is reversed [25]. In the setting of persistent organ failure or poor surgical candidacy, antimicrobial therapy and concurrent percutaneous cholecystostomy should be performed [26].

The role for antibiotic prophylaxis prior to elective laparoscopic cholecystectomy has been studied in prospective randomized trials. Unfortunately the evidence remains insufficient to either support or refute their use in an attempt to reduce surgical site and global infections. This question has not been evaluated for patients undergoing urgent cholecystectomy for acute cholecystitis in any trials however. As a result, consensus guidelines recommend that antibiotic therapy should be started if infection is suspected on the basis of clinical, laboratory, and/or radiographic findings [27]. Treatment should include coverage for the Enterobacteriaceae family (i.e. second generation cephalosporin, or a combination of a quinolone and metronidazole) [4]. Treatment of enterococci is debated. Elderly patients and those with diabetes mellitus or immunosuppressive disorders should receive antibiotics even when infection has not been confirmed. Obtaining aerobic and anaerobic cultures from the bile during surgery is also recommended to guide treatment in complex cases [4].

The role of *routine* intraoperative cholangiography has been evaluated in patients undergoing elective cholecystectomy [28]. Eight randomized trials (1715 patients) were analyzed in a recent systematic review without showing any clear evidence to support its routine use [29]. When this is combined with the fact that there are no randomized studies in patients undergoing cholecystectomy for acute cholecystitis, intraoperative cholangiography should be performed selectively in the setting of concerning pre- and/or intra-operative findings.

#### AC in specific scenarios (jaundice, acalculous acute cholecystitis, pregnancy)

The presence of jaundice should be evaluated with caution because it reflects a wide spectrum of potentially benign and malignant conditions. These include, but are not limited to, CBD obstruction from external compression (cholangiocarcinoma, periampullary cancers, gallbladder cancer), choledocholithiasis, and liver failure (e.g. secondary to sepsis). Although US continues to be the diagnostic gold standard for detecting choledocholithiasis (especially within the distal CBD), MR cholangiography (MRC) may also be useful to define the etiology [30]. The dominant goals in the treatment of patients with choledocholithiasis are three-fold: [1] treat concurrent sepsis, [2] evacuate the CBD, and [3] prevent future recurrences. Although the order of the latter 2 goals is debated on the basis of length of stay, safety and economics, it is clear that ERCP and laparoscopic cholecystectomy represent the 2 dominant therapies [31,32]. Laparoscopic CBD exploration (transcystic or transductal) is also a viable option and has the added benefit of being performed as a single procedure [33,34].

*Acalculous cholecystitis* is an uncommon and serious presentation observed in 5% to 10% of patients with biliary emergencies [35]. It is typically associated with critical illness, immunosuppressive conditions, uncommon pathogens (anaerobes), and/or sepsis [35]. On a global basis, patients with acquired immunodeficiency syndrome (AIDS) continue to represent the most common immunosuppressive cases and are younger, present with elevations in their alkaline phosphatase and serum bilirubin, and may have cytomegalovirus and cryptosporidium associated infections [36]. Other rare causes of acalculous cholecystitis are chemical cholecystitis after hepatic artery infusion, antibiotic-related cholecystitis, and parasites (ascaris). Since patients with acalculous cholecystitis often present with organ dysfunction and are poor surgical candidates, medical treatment is often the therapy of choice, with surgery performed in selected cases (i.e. if cholecystostomy is ineffective) [35].

Pregnant patients carry a higher risk of developing both gallstones and acute cholecystitis than non-pregnant patients [37,38]. Complications of gallstones remain the second most common cause of surgery during pregnancy [39]. Despite this epidemiology, surgery should be avoided during the first (i.e. abortion) and third (i.e. premature delivery) trimesters if possible. Most symptomatic patients treated with nonoperative therapy present with recurrence of their symptoms however. Of this cohort, approximately 30% eventually require surgery during their pregnancy [40].

#### Acute cholangitis

Acute cholangitis is defined as acute inflammation and infection of the biliary tree. This is most commonly a consequence of biliary obstruction followed by bacterial overgrowth within bile [41]. The dominant cause of acute cholangitis is choledocholithiasis, followed by benign biliary stenosis and cancer. Interestingly, a recent report that included 794 patients did not show a significant difference between the incidence of gallstones (50.6%) and malignancies (49.4%) as the main source of obstruction [8,42]. This likely reflects an aging populous. Regardless of the cause or site, obstruction of the biliary tree (most commonly of the common bile duct), leads to an increase in the number of microorganisms followed by a rise in the intraductal pressure of the bile duct. This increases ductal permeability and facilitates translocation of bacteria and their products into the vascular system. This process is highly dependent upon contamination of normally aseptic bile. It is also supported by data that reports 16% of patients undergoing a non-biliary operation, 72% with acute cholangitis, 44% with chronic cholangitis, 50% with acute biliary obstruction, and 90% of patients with choledocholithiasis and jaundice have positive biliary cultures [8].

Patients with cholangitis may present with a wide variety of symptoms from nonspecific findings to severe infection and fatal septic shock. Morbidity and mortality are minimized only via early diagnosis and treatment. Given that Charcot’s triad (jaundice, fever and right upper quadrant peritonitis) has a high specificity (>90%), but is observed in only 18.5% of patients, a high level of suspicion for the diagnosis is essential [3].

This reality is reinforced by the Tokyo guidelines that summarize the diagnosis must include at least [1] signs of systemic inflammation (i.e. fever), [2] cholestasis, and [3] specific findings on imaging [3,5] (Table 2). Current recommendations also grade the severity of cholangitis (I (mild = diagnosis of exclusion), II (moderate = systemic inflammation without organ dysfunction), or III (severe = concurrent dysfunction of at least 1 organ system)). Despite this grading system, most patients (54%) present with grade I disease (only 11% develop grade III).

**Table 2:** **“Severity assessment criteria for acute cholangitis”**

**Grade**	**Definition**
I (mild)	Acute cholangitis does not meet the criteria of “Grade II or III”, representing acute cholangitis at initial diagnosis. Notes
	Patients should have early diagnosis, biliary drainage and/or treatment for etiology, and antimicrobial administration.
	It is recommended that patients with acute cholangitis who do not respond to the initial medical treatment (general supportive care and antimicrobial therapy) undergo early biliary drainage or treatment for etiology.
II (moderate)	Acute cholangitis associated with any two of the following conditions:
	1. Abnormal white blood cell count (>12,000/mm^3^, < 4,000/mm^3^)
	2. High fever (≥39°C)
	3. Age (≥75 years old)
	4. Hyperbilirubinemia (total bilirubin ≥ 5 mg/dL)
	5. Hypoalbuminemia (< STD × 0.7)
III (severe)	Acute cholangitis associated with the onset of dysfunction in at least one of any of the following organs/systems:
	1. Cardiovascular dysfunction defined as hypotension requiring treatment with dopamine ≥ 5 μg/kg per min, or any dose of norepinephrine
	2. Neurological dysfunction defined as decreased level of consciousness
	3. Respiratory dysfunction defined as a PaO2/FiO2 ratio < 300
	4. Renal dysfunction defined as oliguria, creatinine > 2.0 mg/dl
	5. Hepatic dysfunction defined as PT-INR > 1.5
	6. Hematological dysfunction defined as platelet count < 100,000/mm3

STD lower limit of normal value.

Kiriyama S, et al. [5] (with permission).

The treatment of acute cholangitis has shown dramatic improvement over the past decade with a current mortality of less than 30% (i.e. sepsis leading to multiorgan failure) [3]. Patients should receive general medical therapy including NPO, intravenous fluids, antibiotics, and analgesia [27]. In addition to critical care, various techniques for biliary decompression are also mandated [43]. Approaches include endoscopic, percutaneous and/or operative approaches based on the etiology of the cholangitis and patient physiology [44,45]. Medical treatment may be sufficient in selected cases of grade I, but biliary drainage should be considered for all non-responders [46]. This scenario often incorporates postoperative patients. Patients with grade II disease require early endoscopic, percutaneous or emergent operative (T-tube) biliary drainage. These drainage procedures may be definitive for patients with gallstone-associated cholangitis [46]. Although it remains controversial if patients with cancer should undergo a definitive resection concurrent to their emergent decompression procedure, this is generally not recommended due to a lack of complete staging, higher postoperative complications, and known hospital volume-outcome relationships. Patients with cancer should be stabilized, undergo emergency drainage, and be referred for definitive treatment to a high volume HPB center. In the most critically ill patients, a multidisciplinary team of endoscopists, intensive care physicians and sometimes HPB surgeons should participate with the ACS in the management of these patients. The type of biliary drainage selected by the surgeon is particularly important. Patients with hilar cholangiocarcinoma most often benefit from a percutaneous technique (percutaneous transhepatic catheter (PTC) as opposed to endoscopic). ERCP-based attempts at drainage of hilar cholangiocarcinoma are notorious for failing to achieve adequate drainage within the proximal liver and subsequently often develop significant cholangitis due to instrumentation of the biliary system during this attempt. These patients *must* also receive high fidelity cholangiographic information either prior to (MRCP), or concurrent to the insertion of the PTC (i.e. via the PTC itself) to define resectability and reconstruction options. Patients with any periampullary malignancy should undergo ERCP-based stenting as the initial choice.

Patients with grade III cholangitis require admission to the intensive care unit for physiologic support, in addition to general medical treatment [46]. Urgent endoscopic, percutaneous or surgical biliary drainage must also be performed. Considering that these patients are often physiologically unstable, the most rapid and least invasive procedure should be selected (operative intervention should be the last resort given the high associated mortality). Once the cholangitis has resolved, definitive treatment of the etiology (i.e. laparoscopic cholecystectomy for cholelithiasis, resection for cancer) is indicated.

### Severe acute pancreatitis (SAP)

Acute pancreatitis is a common disease that affects approximately 200,000 people per year in the United States [47]. It was classically defined by the Atlanta guidelines in 1992 as “an acute inflammatory process of the pancreas with variable involvement of regional or distant organ/systems and high levels of pancreatic enzyme in blood and/or urine” [48]. Although the differential diagnosis for causes of pancreatitis is broad, choledocholithiasis remains the most common [49]. SAP is observed in approximately 20% of patients, and depends on the presence of organ failure and local complications (necrosis, pseudocyst, and walled off pancreatic necrosis). The dominant definition of SAP includes subsets of patients with organ failure, necrotizing pancreatitis without organ failure, and infected pancreatic necrosis with organ failure [2,50]. Given that this definition considers groups with vastly different prognoses, it has been recently revised by a group of experts [50]. It is now based on local and systemic determinants of severity. The *local determinants* include pancreatic and/or peripancreatic necrosis (P-PN). These 2 entities have been included together and represent all non-viable tissue (solid or semisolid) that does not have a radiologically defined wall. Thus, pancreatic necrosis is defined as any area of non-enhancement on contrast-enhanced CT and peripancreatic necrosis includes every heterogeneous peripancreatic collection on CT until proven otherwise. P-PN may be considered infected when gas bubbles are observed on CT scan, or when a culture obtained by percutaneous or surgical techniques is positive [50]. It should be noted that the routine use of broad-spectrum prophylactic antibiotics has altered the bacteriology of secondary pancreatic infection in severe acute pancreatitis, from predominantly gram-negative to gram-positive bacteria without changing the rate of beta-lactam resistance or fungal super-infection [51]. *Systemic determinants* refer to distant organ failure, which is defined for 3 systems on the basis of the worst measurement in a 24-hour period or when the creatinine is ≥ 2 mg/dl, PaO_2_/FiO_2_ is ≤ 300, or inotropic agents are required. *Transient failure* is defined as failure of the same organ/system for less than 48 hours, while *persistent failure* implies more than 48 hours. Based on this new consensus grading, acute pancreatitis is described as [1] *mild (*P-PN and organ failure are absent), [2] *moderate* (sterile P-PN and/or transient organ failure), [3] *severe* (either infected P-PN or persistent organ failure), or [4] *critical* (infected P-PN with persistent organ failure) [50].

Despite guideline recommendations, every patient should receive personalized treatment based on the severity of their disease and the direction of a multidisciplinary team. The surgical treatment of patients with SAP has evolved dramatically and includes open, laparoscopic, radiologic, and endoscopic techniques of debridement and drainage [52,53]. These approaches may be used alone, or in combination [54]. Despite this long list of potential techniques to remove necrotic tissue, the more dominant issue for the ACS surgeon is one of timing. It is clear that early debridement increases blood loss, morbidity, the number of operative interventions, and mortality across all groups of patients with SAP [55]. More specifically, almost every patient should be physiologically supported without major intervention until the 28-day mark. Patients with SAP follow a predictable pattern of early SIRS and potentially multi-organ failure as a result. This interval observation is often misinterpreted as sepsis requiring treatment with antimicrobial therapy. Within the first 7 to 10 days, very few of these patients have infected necrosis (and therefore require antibiotics). The most common exceptions to this rule of delayed (28-day) intervention remains patients with concurrent ischemia of the bowel or gallbladder [56]. As a result, it is ischemia of these 2 organs that must be ruled out if a critically ill patient decompensates, as opposed to focusing on the status of the pancreas itself (infected or not).

Once the patient is stabilized and the pancreatic necrosum is mature, operative therapies may include both minimally invasive (laparoscopic cystgastrostomies and debridements, utilization of percutaneous drains as access guides for rigid scope debridement, step-up procedures, endoscopic transmural debridements) and open (transperitoneal, retroperitoneal-flank) approaches [47,57-59]. The best choice amongst these options is based on patient anatomy and the specific location(s) of the necrosum.

The recent publication popularity in percutaneous techniques as treatment for pancreatic necrosis deserves specific mention. CT-guided drainage followed by repeated irrigation procedures in the context of ever increasingly larger drains placed by involved and committed radiologists may improve the clinical course in up to 75% of patients. It has also been shown to resolve the necrotic collection in 45% of cases. This technique is incredibly labor intensive however, and therefore not available in most centers.

In addition to the role of surgery, significant literature exists with reference to the role of prophylactic antibiotics, early ERCP decompression in persistent biliary pancreatitis [60,61], type of nutrition [62], role of octreotide, and probiotic prophylaxis. The use of prophylactic antibiotics has been evaluated in numerous underpowered prospective randomized trials and meta-analyses [63-66]. These 7 RCTs are best summarized by stating that there is no good evidence to support the routine use of prophylactic antibiotics in patients with SAP [67,68]. Furthermore, the general recommendation is to stop all antibiotics if they have been previously started in this scenario.

The optimal type of nutrition (enteral vs. parenteral) has also been evaluated in multiple prospective randomized trials. A systematic review that included 348 patients from 8 trials showed that enteral nutrition decreased the risk of death (OR 0.50, 95% CI 0.28 to 0.91), multiple organ failure (OR 0.55, 95% CI 0.37 to 0.81), systemic infection (OR 0.39, 95% CI 0.23 to 0.65), operative interventions (OR 0.44, 95% CI 0.29 to 0.67), local septic complications (OR 0.74, 95% CI 0.40 to 1.35) and length of hospital stay (reduced by 2.4 days) [69]. More importantly, in patients with SAP, enteral nutrition decreased the risk of death (RR = 0.18, 95% CI 0.06 to 0.58) and multiple organ failure (RR = 0.46, 95% CI 0.16 to 1.29), suggesting that patients should receive enteral over parenteral nutrition [69]. In many patients, regardless of the extent of necrosis on CT, oral ingestion of a regular diet is well tolerated. If this fails, progression to nasogastric followed by nasojejunal tube feeding as needed is recommended.

The role of early ERCP in the context of choledocholithiasis has also been evaluated in prospective studies [61,70-74]. Amongst 153 patients in a multicenter prospective study, patients were divided into 2 groups (with and without signs of cholestasis) [75]. Although ERCP was associated with fewer complications than the observation group (25% vs. 54%, p = 0.02) in patients with signs of cholestasis, mortality was not significantly lower (6% vs. 15%, p = 0.2). Additionally, ERCP neither reduced complications (45% vs. 41%, p = 0.8) nor mortality (14% vs. 17%, P = 0.7) in patients without cholestasis, suggesting that ERCP should be indicated only in selected patients with *persistent* cholestasis [61,74].

Finally, trials evaluating probiotic use reported that prophylaxis did not reduce the risk of infectious complications, but actually increased the risk of mortality in patients with predicted SAP [76]. Similarly, another trial of 302 patients with moderate to SAP who received either octreotide or placebo had similar rates of mortality, complications, duration of pain, surgical interventions, and length of hospital stay. This finding suggests that octreotide should not be used in SAP [77].

### Non-traumatic injuries

#### Iatrogenic bile duct injuries (BDI)

BDIs can be physically and psychologically challenging errors for both the injurious surgeon, as well as their colleague asked to repair the injury [78]. Although a detailed description of BDIs and their reconstructions is beyond the scope of this review, it is important to consider this diagnosis, as the ACS surgeon is often called as the initial consultant by a surgeon in need. Based on large population studies, the rate of BDIs approaches 0.4% using laparoscopic techniques and 0.1% with open approaches [79]. Most BDIs are not recognized intraoperatively and instead patients return to the hospital with complaints of nausea, vomiting, abdominal discomfort and potentially obstructive biliary symptoms [80]. The ACS surgeon must hold diagnoses of a BDI and/or biloma high in their differential diagnosis when assessing these postoperative patients. Intraoperative diagnoses of BDIs should be suspected in cases of extensive inflammation, severely contracted gallbladders, unexpected bleeding that requires multiple clips for control, abnormal anatomy, bile within the operative field, or difficultly in defining the triangle of Calot, sulcus of Rouvier, median umbilical fissure, hepatic artery and critical view of safety [81]. If an injury is suspected, but not clearly evident, an intraoperative cholangiogram should be performed to evaluate the biliary tree as a first assessment [82]. Given the tremendous frequency of general surgeons to misinterpret intraoperative cholangiograms however, more than one surgeon with experience and/or a radiologist is ideal. If doubt persists, consultation with an HPB surgeon experienced in BDI reconstruction prior to conversion to an open procedure is highly recommended. The status of the right hepatic artery (90%) in addition to the proximity of the injury and the quality/loss of ductal tissue will define recommendations from the referral surgeon [83]. If the surgical team does not have the expertise to repair the bile duct, 2 closed suction drains should be placed within the gallbladder fossa to manage a potential bile leak, followed by immediate referral and/or transfer. Intentional occlusion (clip or tie) of a transected common bile duct during the index operation is not helpful due to a lack of subsequent proximal dilation, as well as necrosis of the duct leading to a more proximal injury.

Delayed presentation of a BDI should be suspected by the ACS in patients who present with nausea, vomiting, fever and persistent abdominal pain that does not decrease with regular analgesics. Liver function tests may be abnormally elevated not only due to obstruction, but also because of a biloma. Patients should be studied immediately with an abdominal US and/or CT to define the presence of collections or abnormal free fluid. It is important to note that the absence of fluid does not exclude the occurrence of a BDI. Any fluid collection should be drained with a percutaneous approach. The biliary tree, and specifically the level of injury, may then be defined with an MRC and/or ERCP [84,85]. In scenarios where the posterior sector has been isolated or there has been a complete common ductal transection, retrograde drain cholangiograms and percutaneous transhepatic cholangiograms (and catheters) are required respectively [86].

It should be noted that in all BDI patients, the cause of sepsis may be multifold: [1] intraabdominal collections/biloma (usually related to the gallbladder bed), [2] biliperitoneum, [3] cholangitis (when the bile duct has been completely transected and clipped), and [4] liver necrosis/failure when the BDI is associated with a vascular injury [87]. Since the specific cause of sepsis is usually unknown at the time of presentation, all critically ill patients should receive immediate fluid resuscitation and antibiotics. A CT scan should be obtained to assess the etiology of sepsis. Blood and intraabdominal cultures are mandatory to guide the therapy.

#### Duodenal perforation following ERCP

ERCP is a diagnostic and therapeutic procedure that is employed in a wide range of benign and malignant diseases. It has been described that approximately 10% to 15% of patients sustain procedural complications. Although pancreatitis remains the most common, duodenal perforations occur in approximately 1% of patients [88-90]. Unfortunately the mortality associated with these perforations is as high as 20% depending on the series [88]. Treatment depends entirely on patient clinical presentation, location of the perforation, and the initial indication for the ERCP [91,92]. Injuries are classified as type I (created in the lateral wall of duodenum or jejunum during the ERCP scope’s “long view”) or type II (small perforations of the ampulla of Vater including the periampullary tissues) [93]. Unlike BDIs during laparoscopic cholecystectomy, most cases (74%) of ERCP perforations are detected during the ERCP itself [94]. The majority of type I injuries (81%) require surgical management (duodenal wall repair and periduodenal drainage with occasional Roux-en-Y duodenojejunostomy), whereas most type II (97%) injuries are treated conservatively (antibiotic therapy and drainage of retroperitoneal collections). It should be noted however that failure of medical management (including percutaneous drainage) is typical in scenarios of significant retroperitoneal fluid [95]. In contrast, patients with significant retroperitoneal air and very little fluid often recover well with nonoperative therapies (antibiotics, NPO).

In conclusion, HPB emergencies for the ACS surgeon are broad in nature and presentation. Although the appropriate treatments can be complex and require multimodality care, they are each approached similarly in the acute phase. This initial assessment involves both diagnosis, as well as treatment with resuscitation, antimicrobial therapy and multidisciplinary input. By approaching HPB emergencies in this manner, the ACS surgeon will be well equipped to deal with all patients.
